# Household cooking frequency and diet quality are mediated by food shopping behaviors among U.S. African-American adults: A NHANES analysis

**DOI:** 10.1371/journal.pone.0326481

**Published:** 2025-06-24

**Authors:** Nicole Farmer, Narjis Kazmi, Kristina Franklin, Li Yang, Tiffany M. Powell-Wiley, Gwenyth R. Wallen

**Affiliations:** 1 Translational Biobehavioral and Health Promotion Branch, National Institutes of Health, Clinical Center, Bethesda, Maryland, United States of America; 2 Social Determinants of Obesity and Cardiovascular Risk Laboratory, National Heart, Lung and, Blood Institute, Bethesda, Maryland, United States of America; 3 Intramural Research Program, National Institute on Minority Health and Health Disparities, Bethesda, Maryland, United States of America; University of Sannio, ITALY

## Abstract

Among U.S. adults who self-identified as African-American, frequent household cooking is related to better dietary quality and adherence to U.S. dietary guidelines, as measured by the Healthy Eating Index. However, African-Americans often reside in commercially disinvested areas with limited access to major food retailers. This study examined whether food shopping behaviors—travel time to grocery store and frequency of major food shopping—affect the relationship between cooking frequency and diet quality, potentially influencing community nutrition education outcomes. Using data from 2,434 non-Hispanic Black adults (≥ 18 years) from the 2007–2010 NHANES cycles, we performed linear regression analysis with Complex Sample General Linear Models (CSGLM). The Healthy Eating Index 2010 measured daily and dinner dietary quality. Mediation analysis was conducted to evaluate if food shopping behaviors are involved in potential causal pathways. Results showed that major food shopping frequency, but not travel time to a grocery store, was significantly associated with daily diet quality and cooking frequency (p < 0.001). Mediation analysis revealed that food shopping frequency significantly mediated the relationship between cooking frequency and dinner quality (52.44% mediating effect). When stratified by food security status, shopping frequency was only a significant mediator of cooking frequency and diet quality for those with full or marginal food security (64.89% mediating effect). For food-insecure individuals, major food shopping did not mediate the cooking-diet quality relationship. These findings suggest that food shopping frequency is a critical factor when assessing the link between cooking frequency and diet quality, and may inform factors to optimize food shopping behaviors within community nutrition education programs among those experiencing food-insecurity in the U.S.

## Introduction

Lack of an optimal diet is common among U.S. adults, [1] and is a primary risk factor for many chronic diseases [2–4]. Diet quality is improved from food choices that include fruits, vegetables, and whole grains. Eating at home and preparing meals at home (cooking) are two diet behaviors that are directly associated with a more optimal diet quality. To accomplish improvements in diet quality and to possibly engage more in behaviors that promote optimal diet, such as cooking, individuals have to procure or shop for their foods and purchase the ingredients and food items that compose an optimal diet quality. The retail food environments utilized by individuals is a significant factor to consider in efforts to improve diet quality, as a large majority of daily energy from food consumed is from foods purchased in food retail stores [5,6]. Information about how individuals utilize food environments can be obtained from investigating food shopping behaviors, such as frequency of going major food shopping and purchasing habits. Interestingly, how food shopping behaviors may interact with other diet behaviors related to a more optimal diet quality, such as cooking, has not been widely reported.

Individuals’ dietary behaviors, food shopping behaviors, and health outcomes are embedded within social, economic and physical environments [7]. Shopping behaviors are influenced by the available selection of shopping destinations, in-store marketing, food availability, affordability and food quality [8,9]. As these factors are often linked to the food environments that individuals shop within, food shopping behaviors are inextricably linked within their social, economic and physical environment [10,11]. Examining food shopping behaviors provides additional insight into the use of food environments that cannot be captured through utilizing distance-based proxies of accessibility because determination of food purchases extends beyond access and immediate environments [12]. Product prices, available income on food, nutrition knowledge, and food preferences are reported as more important determinants than access for consumers’ choices on food purchase [13]. Therefore, understanding food shopping behaviors can be an important measure of how an individual utilizes their proximal (immediate residential) food environments and food environments outside of their immediate residential area [14,15].

For many individuals, one specific food shopping behavior, frequency of going food shopping, is reported as directly associated with diet quality. Prior studies have found a direct relationship with frequent food shopping at grocery stores and purchase and consumption of fruits and vegetables [12,16–19]. In contrast, perceived travel time or distance to a grocery store, another food shopping behavior, serves as a measure of geographic access and may not necessarily provide information on an individual’s use of food environments outside of their immediate area [20]. A study of U.S. households found that on average, Americans usual food shopping store was over 1 mile away from their nearest supermarket or supercenter [13]. In further support of this evidence, self-reported distance nor time to nearest grocery store was associated with fruit and vegetable intake, body mass index or sugar sweetened beverages [21]. Suggesting that geographic access, although important, may not be sufficient to understand the multiple factors that connect retail food environments and diet outcomes [21]*.*

Over the past decade, interest in evaluating cooking and meal preparation frequency as a diet behavior that encourages optimal diet quality has emerged. Food shopping behaviors, such as frequency and purchasing patterns, may drive an individual’s ability to cook with fresh ingredients versus boxed or processed ingredients, which would not optimize diet quality or health outcomes. Understanding this interaction with food shopping and cooking could lead to insight into how to promote healthier methods for both behaviors. In a study of young adults, increased participation in grocery shopping and dinner preparation was associated with healthier dietary habits, and engaging in shopping at least once weekly was associated with increased vegetable and fruit consumption [22]. Additionally, more-frequent trips and fewer small trips were associated with healthier purchasing for fruit and vegetables and less-healthy foods/beverages [18]. The interaction between food shopping and cooking cannot exclude determinants related to income. A survey of U.S. adults found that low-income households were more likely to cook breakfast and lunch, but not dinner, whereas higher income households were more likely to cook dinner but with more processed food ingredient items [23].

Investigating the role of the food shopping behaviors among individuals who may predominantly or disproportionately live in geographic areas with adverse food environments could aid in identifying strategies employed by these individuals to facilitate healthier food choices. Self-identified African-Americans are a group of racially categorized individuals disproportionately affected by both chronic disease and adverse food environments [16,24–26]. A study of African-American women found that those shopping primarily at supermarkets and specialty stores consumed more fruits and vegetables on average than those who shopped primarily at independent grocers [27]. And a study of low-income African-American adults found that frequent corner-store shoppers procured unhealthy foods more frequently than frequent supermarket shoppers [28]. Thus, frequency of shopping and shopping primarily at a supermarket, food retailer that have broader selections of food items, for African-American women is a significant factor on diet quality. Further, an interrelationship between frequent food shopping and distance to a grocery store, as well as access to personal transportation, was reported from a study of predominately African-American households [29]. Of interest, among a survey of households in Texas, African American families reported shopping for food least frequently compared to other ethnic groups [20]. Related to cooking, we have previously reported that among African-American adults higher cooking frequency at the household level is directly associated with objective dietary quality among middle income households. The influence of food shopping behaviors on this association has yet to be explored among a nationally representative sample of U.S. self-identified African-American adults. Given this interconnection, it is important to consider these factors when evaluating the potential relationship between home cooking frequency and diet quality. In our prior analysis, income stratification showed that low-income households did not have a significant relationship between cooking frequency and diet quality.

We therefore sought to fill existing gaps in the literature by using a large, representative sample of African-American adults participating in the National Health and Nutrition Examination Survey (NHANES) and examine: 1) relationship with household dinner cooking frequency and food shopping behaviors, 2) the relationship between food shopping behaviors and diet quality at dinner and throughout the day, 3) determine if food shopping behaviors are mediators of the relationship between household dinner cooking frequency and diet quality, and if this mediation varied by food security status or household income level. We hypothesize that there will be a direct relationship between cooking frequency, dietary quality and food shopping behaviors. We further hypothesize, that any mediation from shopping behaviors would differ by food security status and income level, as it is expected that food environments for these factors would differ across these variables.

## Materials and methods

### Data and sample

NHANES (National Health and Nutrition Examination Survey) is a cross-sectional survey designed to monitor the health and nutritional status of the civilian noninstitutionalized U.S. population [30]. Due to the NHANES complex sample design, sample weights and sample design variables were used to obtain unbiased estimates representative of the U.S. population [31]. To calculate national estimates and standard errors, data files from the 2007–08 and 2009–10 cycles were combined [30,31] to get the four-year weights. Cooking frequency, our independent variable of interest, was only fielded during the 2007–2008 and 2009–2010 cycles within the Consumer Behavior data section of NHANES [30,31].

The sample population of interest for this analysis is non-Hispanic Black adults (age ≥ 19 years of age). In accordance with the population definitions used in NHANES, the term non-Hispanic Black represents non-Hispanic Blacks (born in the U.S.) and individuals of African descent living in the U.S. (not born in the U.S.). Of the 20,686 participants aged 19 years or older who participated in the NHANES cycles 2007–2008 and 2009–2010, cooking frequency data were available for 20,375. Participants who self-identified as Non-Hispanic Black, which is inclusive of self-identified African-Americans, were selected from this number to yield a total of 2434 participants for this analysis.

### Study variables

Household cooking frequency was evaluated as the independent variable using the NHANES question, “In the past 7 days, how many times did you or someone in your household cook dinner at home?” The self-reported frequency of cooking dinner at home, was divided into three categories: 0–1 dinner cooked per week (‘low’), 2–5 (‘sometimes’) and 6–7 (‘always’) as previously reported [32]. The categories for cooking frequency were selected based on prior literature and to provide comparison of our results with prior reports [32–34]. Responses were excluded that provided an answer > 7 for cooking dinner frequency question.

Indicators of food shopping behaviors were measured using the available variables of travel time to a grocery store and major food shopping frequency. To determine the travel time co-variate, the question, “How much time does it usually take you to get to the grocery store for food shopping?” was used from the NHANES consumer behavior interview. All responses reflected a one-way trip to the grocery store. If respondents mentioned more than one grocery store, they were asked to report the time to get to the grocery store they go to most often. Frequency of major food shopping was determined using the NHANES consumer behavior question, “How often do you or someone in your family do the major food shopping for yourself/your family? Respondents were told to not include frequencies when only a few food items were purchased. Responses were recoded as in Pitts et al, [35] to create the following variable categories: one or more times a week; once every two weeks or once a month; and rarely any. Although the cited literature has utilized grocery shopping frequency, the NHANES question wording is inclusive of grocery store shopping as it states major food shopping. For mediation analysis, the frequency of major food shopping was recoded to two groups: one or more times a week and less than once per week.

Objective dietary data was the dependent variable of interest. Dietary recall data were used to calculate the objective diet quality measurement, Healthy Eating Index 2010 (HEI-10), a diet quality index that measures conformance to federal dietary guidelines [36]. Key features of HEI are that diet quality is assessed from the perspectives of adequacy and moderation; the scoring standards are density-based, such that the relative mix of foods is evaluated; and the standards for the maximum scores are the easiest to achieve recommendations among those that vary by energy level, sex, and/or age [37]. Total scores for HEI range from 0 to 100 and higher scores indicate better diet quality. A score above 80 indicates good diet quality, a score of 51–80 indicates a need for improvement, and a score of 50 or below indicates a poor diet quality. Total nutrient intakes from 24-hour dietary data are used to determine daily HEI, dinner HEI and component scores, as described in Farmer et al. [33]. Dietary recall was collected from validated NHANES 24-hour dietary recall interviews. The population ratio method was used for calculation of HEI-10 consistent with recommendations provided by Kirkpatrick et al. [38]

Food security was determined using the NHANES calculated ‘Household food security category’ variable created from the US Food Security Survey Module (US FSSM) [39]. The household food security category (FSDHH) variable offers four response levels: full food security, marginal food security, low food security, and very low food security. For mediation analysis, the categories of food security were recoded to two groups: full or marginal security and low or very low security. Birthplace status was determined by the dichotomous variables: born in the U.S. or Washington, D.C. or not born in the U.S. or Washington, D.C.. Other demographic and SES variables selected were gender and number of people in household. Poverty income ratios were recoded after analysis of data frequencies into categories as presented in Powell-Wiley et al. [40]. Work status was calculated using NHANES occupation variables ‘type of work done last week’ and ‘hours worked last week at all jobs’ as in Wolfson and Bleich. [35]. Marital status and education were calculated using NHANES variables, and after analysis of data frequencies as described in Farmer et al. [33].

### Statistical analysis

All data analyses were performed using SPSS Complex Samples IBM SPSS 24 (IBM Corp, Armonk, NY, USA) [41]and R (version 4.3.2) Lavaan package (Lavaan 0.6–15) [42]. The four-year weights and the masked variance units for strata (sdmvstra) and PSU/cluster (sdmvpsu) were used for all complex sample analyses in this paper. Appropriate descriptive statistics (frequencies and percentages for categorical variable and means and standard errors for continuous variables) were used to describe the whole sample and each cooking frequency group. Complex Sample General Linear Model (CSGLM) were used to evaluate the relationship between meal purchases, consumption of convenient foods, food spending, and diet quality. Co-variates were included in linear regression models if demographic, socioeconomic, and dietary behavior co-variates from bivariate analysis were significantly related to the main dependent variable in each model. Unweighted simple mediation analyses were conducted using the R Lavaan package to test whether major grocery shopping frequency mediated the relationship between cooking frequency and diet quality. Using the R Lavaan package, a serial of regression models were run to test three mediation paths. Path *a* tested the effect of cooking frequency on major grocery shopping frequency. Path *b* examined the effect of major grocery shopping frequency on diet quality, while controlling for cooking frequency. Path *c’* assessed the direct effect of cooking frequency on diet quality after accounting for major grocery shopping frequency. The indirect effect, calculated as the product of paths a and b, represents the extent to which major grocery shopping frequency explains the relationship between cooking frequency and diet quality. A p-value less than 0.05 was considered statistically significant.

## Results and discussion

A total sample of 2,336 non-Hispanic Black adults (weighted U.S. population 24,949,641.22) were included in this analysis. Characteristics of the sample adults based on individual and food environment variables by cooking group frequency are shown in Table 1. The mean age was 43.8 years (±0.51), with 55.2% (±0.9%) of the population identifying as female and 56.0% (±1.4%) reporting that they were never or previously married. In terms of socioeconomic status, 44.1% (±1.4%) reported not working, 51.0% (±2.1%) had household income in the highest poverty/income ratio, with 48.8% completing high school as their highest level of education. Consistent with published literature evaluating weekly cooking frequency of dinner among non-Hispanic Blacks,1 the majority of non-Hispanic Black adults reported a cooking frequency within the sometimes cook group (51.4%) compared to always (35.5%) and low (13.6%) cooking frequency groups. Unadjusted complex sample regression analysis showed that frequency of major food shopping was significantly related to both HEI-10 daily scores (R^2^ = 0.009, p < 0.001), and HEI-dinner score (R^2^ = 0.002 p = .019). Travel time to grocery store was not significantly associated with either variable (p = .413 and p = .928, respectively). Chi-square analysis showed that there was significant relationship between major food shopping frequency and food security status (adjusted F(3.2, 93.5) =3.75, p = 0.012), as shown in Supplement Table 1. When adjusted for age, gender, birthplace, poverty-to-income ratio and education, major food shopping was significantly associated with HEI-10 daily (F (2,28) = 4.372,p = 0.022). Participants who reported major grocery shopping *one or more times a week* had significantly higher HEI-10 daily scores compared to those who shopped *once every two weeks or once a month* (F (1,29) = 8.544, p = 0.007). However, there were no statistically significant differences between those who reported *rarely any* major food shopping compared to those who shopped *one or more times a week* (F(1, 29) = 0.228, p = 0.636), or those who shopped *once every two weeks or once a month* (F(1, 29) = 0.203, p = 0.656). Unadjusted models showed similar results that major grocery shopping frequency was significantly associated with HEI-10 daily (F (2,28) = 15.996, p < 0.001). Participants who reported major grocery shopping once or more times a week had significantly higher HEI-10 daily scores compared to those who shopped once every two weeks or once a month (F (1, 29) = 22.787, p < 0.001). The adjusted model for major food shopping frequency was not associated with HEI-10 dinner score (p = 0.390).

**Table 1:** Demographic, socioeconomic characteristics across cooking frequency categories among Non-Hispanic Black (NHB) adults, U.S. NHANES, 2007–2010.

		Frequency of cooking dinner at home each week	
	Total	Low cook (0–1)	Sometimes cook (2–5)	Always cook (6–7)	p- value
Observations	2336	318	1147	871	
Weighted population	24949641.2	3384188.2	12712694.1	8852759.0	
Total U.S. NHB Population (%)	100%	13.6%	51.0%	35.5%	
Age (Mean, SE)	43.8 (0.51)	42.6 (0.78)	42.9 (0.59)	46.0 (0.97)	0.003*
Gender (%, SE)					0.84
Male (n = 1153)	44.8% (0.9%)	13.9% (1.3%)	50.5% (2.0%)	35.6% (1.5%)	
Female (n = 1210)	55.2% (0.9%)	13.3% (1.4%)	51.3% (1.7%)	35.4% (1.6%)	
Country of birth (%, SE)					0.20
Born in 50 US States or Washington, DC (n = 2126)	89.4% (2.0%)	14.2% (1.2%)	51% (1.6%)	34.8% (1.5%)	
Not Born in U.S. (n = 237)	10.6% (2.0%)	8.1% (1.9%)	50.9% (6.5%)	41% (5.7%)	
Marital Status (%, SE)					<.001*
Married or living with a partner (n = 1027)	44.0% (1.4%)	8.0% (0.9%)	52.7% (1.7%)	39.2% (1.5%)	
Previously or never married (n = 1249)	56.0% (1.4%)	17.4% (1.6%)	50.0% (2.2%)	32.6% (1.8%)	
Employment (%, SE)					<.001*
Not working (n = 1181)	44.1% (1.4%)	13.7% (1.5%)	44.2% (2.1%)	42.1% (2.1)	
Part-time (1–34 hours) (n = 302)	13.9% (0.9%)	13.5% (2.1%)	50.7% (2.7%)	35.7% (2.8%)	
Full-time (>/ = 35 hours) (n = 879)	42.0% (1.2%)	13.4% (1.6)	58.3% (2.7%)	28.3% (2.4%)	
Household Size (%, SE)					0.01*
1-3 (n = 1503)	61.2% (2.3%)	17.3% (1.7%)	50.3% (2.2%)	32.3% (2.2%)	
≥ 4 (n = 860)	38.8% (2.3%)	7.5% (1.7%)	52.0% (3.9%)	40.5% (3.2%)	
Adult food security (%, SE)					0.02*
Full (n = 1559)	63.6% (2.2%)	15.7% (1.5%)	50.7% (2.1%)	33.6% (1.9%)	
Marginal (n = 326)	15.2% (1.4%)	8.9% (1.6%)	53.2% (3.2%)	37.9% (3.1%)	
Very Low/Low (n = 462)	21.3% (1.5%)	10.6% (1.4%)	49.9% (3.0%)	39.6% (2.5%)	
Income to Poverty Ratio (%, SE)					0.02*
<130% lowest (n = 687)	32.1% (1.9%)	13.7% (2.0%)	45.2% (3.1%)	41.1% (3.2%)	
131%−185% middle (n = 361)	16.9% (1.0%)	12.7% (2.5%)	49.1% (2.5%)	38.2% (3.5%)	
>185% highest (n = 1089)	51.0% (2.1%)	13.3% (1.2%)	55.9% (2.0%)	30.8% (1.7%)	
Education (%, SE)					0.07
Less than 9th grade (n = 134)	4.1% (0.5%)	10.1% (4.0%)	46.2% (6.6%)	43.7% (7.7%)	
9-12th grade (n = 1114)	48.8% (1.4%)	13.5% (1.4%)	47.9% (1.9%)	38.6% (2.1%)	
Some college or Associates’ degree (n = 701)	32.0% (1.0%)	13.4% (1.6%)	56.6% (1.9%)	30.0% (1.7%)	
College graduate or above (n = 329)	15.1% (1.0%)	13.3% (2.1%)	52.0% (3.3%)	34.7% (3.3%)	
WIC (%, SE)					0.25
Received (n = 235)	15.0% (1.4%)	7.4% (2.5%)	52.4% (4.3%)	40.2% (4.0%)	
Not received (n = 1400)	85.0% (1.4%)	12.3% (1.3%)	52.9% (2.0%)	34.8% (1.6%)	
SNAP (%, SE)					0.25
Benefits received (n = 621)	70.6% (3.4%)	11.8% (2.1%)	45.7% (3.2%)	42.5% (3.2%)	
Not received (n = 260)	29.4% (3.4%)	12.6% (2.5%)	54.5% (4.2%)	32.9% (4.7%)	
Time to grocery store (mean minutes, SE)	15.05 (0.678)	13.54 (1.347)	14.26 (0.857)	16.81 (1.627)	0.24
Major food shopping frequency					<.001*
One or more times a week (n = 787)	33.3% (1.5%)	7.2% (1.0%)	50.1% (2.9%)	42.7% (2.7%)	
Once every 2 weeks or a month (n = 1477)	64.0% (1.5%)	15.3% (1.5%)	52.2% (1.6%)	32.5% (1.6%)	
Rarely any (n = 74)	2.7% (0.5%)	50.5% (7.5%)	32.9% (7.7%)	16.6% (5.5%)	
HEI2010 total score (n = 2242)	46.58 (0.45)	43.96 (0.98)	46.76 (0.65)	47.20 (0.58)	0.023*
HEI2010 dinner score (n = 2027)	40.90 (0.35)	39.11 (0.84)	41.13 (0.51)	41.23 (0.65)	0.170

Abbreviations: WIC, Special Supplemental Nutrition Program for Women, Infants, and Children.

SNAP, Supplemental Nutrition Assistance Program for low-income individuals/families.

**Fig 1:**
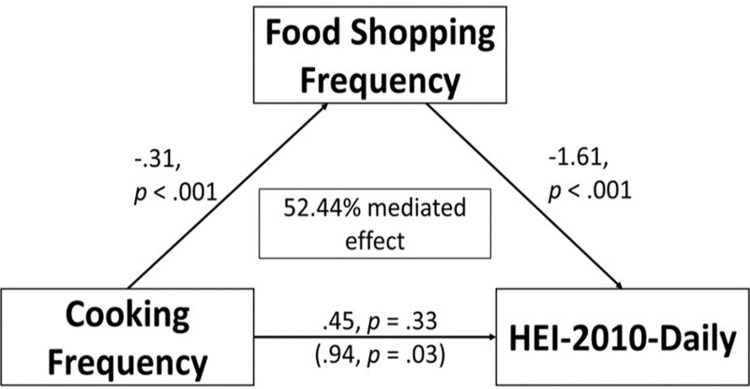
Mediating Effects of Food Shopping Frequency on the Relationship Between Cooking Frequency and HEI-2010-Daily Across the Total Sample (n ** = 2,336)**.

**Fig 2:**
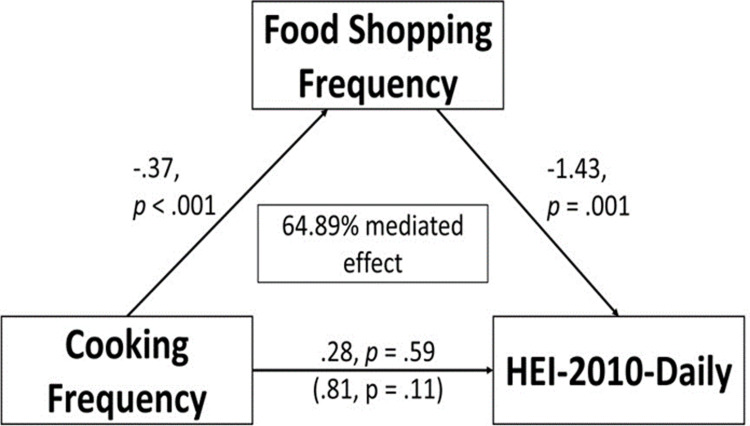
Mediating effects of food shopping frequency on the relationship between cooking frequency and HEI-2010-Daily among individuals with full or marginal food security (n ** = 1772)**.

**Fig 3:**
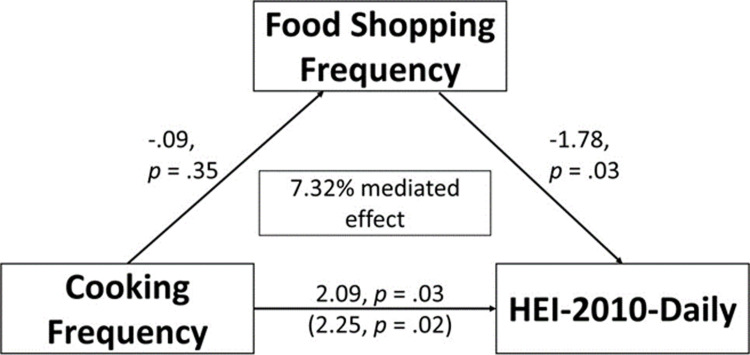
Mediating effects of food shopping frequency on the relationship between cooking frequency and HEI-2010-Daily among individuals with low/very low food security (n ** = 440)**.

Mediation analysis showed a significant *a* path, with cooking frequency negatively predicting food shopping frequency (β = −0.308, s.e. = 0.043, p < 0.001), and a significant *b* path, with food shopping frequency negatively predicting HEI-10 daily (β = −1.609, s.e. = 0.389, p < 0.001). The indirect effect of cooking frequency on HEI-daily through shopping frequency was also significant (β = 0.495, s.e. = 0.139, p < .001), indicating that shopping frequency significantly mediates the relationship between cooking frequency and HEI-10 daily. However, the direct effect (c path) was not significant (β = 0.449, s.e. = 0.457, p = 0.326). These findings from the indirect path suggest 52.44% of the total effect of cooking frequency on HEI-10 daily occurs through shopping frequency (Fig 1). In comparison, when stratified by food security status, the indirect effect of cooking frequency on HEI-10 daily through food shopping frequency only remained significant among individuals with either full or marginal food security (β = 0.65, s.e. = 0.246, p = .008), indicating that 64.89% of the total effect of cooking frequency on HEI-10 daily occurs through shopping frequency among individuals with either full or marginal food security (Fig 2). This result serves as an indication that food shopping frequency is a key mechanism through which cooking frequency at home influences overall daily diet quality for those with full or marginal food security. While for individuals living with low or very low food security, the indirect effect of cooking frequency on HEI-10 daily through frequency of major food shopping was not significant (β = 0.165,s.e. = 0.194, p = 0.393) (Fig 3). A sensitivity analysis related to household income stratification showed that stratified mediation by poverty-income ratio were similar to that of food security status and are presented in Supplemental Fig 1.

### Summary of findings

Food shopping behaviors can be a major determinant of the type of foods available at home, to cook and prepare. Understanding food shopping behaviors may elucidate the influences on connections between preparation, consumption and diet quality [20].We previously reported that cooking frequency is positively associated with objective diet quality [32] but potential facilitators or barriers within the food environment that may mediate this association were not included. In the current analysis, and consistent with our hypothesis, we showed that food shopping frequency, as one metric of food environment utilization, was a significant mediator between cooking frequency and dietary quality. However, the mediation was dependent on income and food security status. For interventions that focus on cooking as a dietary behavior, our findings have important implications to measure food shopping frequency or to design studies to facilitate food shopping to assist with diet quality optimization.

Our overall finding that major food shopping frequency is associated with diet quality is consistent with the previous literature [12,17,18,29]. Gustault et al. found that fresh fruit and vegetable consumption was mediated by the frequency of food shopping trips [17]. Among a survey of 832 households, Liese et al. found that frequency of grocery shopping was the only factor acting as an independent, statistically significant factor on fruit and vegetable intake [12]. Similarly, among a large sample of households from the United Kingdom, Pechey and Monsivais [18] found that more frequent trips were associated with healthier purchasing at either high price or low price supermarkets. Thus, taken our findings with that of prior ones, it appears that measuring food shopping frequency is an important variable when considering diet quality. Yet, regression analyses showed that individuals who only reported food shopping frequency once every two weeks or a month had lower HEI daily scores than those in the rarely any food shopping category. Types of food stores used for shopping and food expenditures may represent additional factors that would explain how people who report rare food shopping frequency may still have higher diet quality than those who reported shopping once every two weeks or a month. Although daily diet quality was associated with food shopping frequency, we did not find a significant relationship with dinner diet quality. Dinner diet quality was included in our analysis as dinner is the meal that is most often cooked at home [33]. The lack of association may be due to less variation in dinner choices compared to meals throughout the rest of the day or could represent that the habitual purchasing habits related to food shopping frequency may influence other meals and represent overall food choices outside of what is cooked at home [12,17,18,29].

For food shopping frequency to lead to diet quality it seems logical to state that some grounded facts must allow for this association to occur. These can include availability of fruits and vegetables at the food retail location(s), acceptable costs for these food items and accessible location(s) of the retailers. This last factor may serve as an explanatory factor for not identifying an association with time or distance to a grocery store and diet quality, especially when geographic access offers limited retail or transportation options [36]. In our analysis, we did not find a relationship between perceived distance to grocery store and diet quality or cooking frequency. Our results may come from the fact that questions about personal access to a vehicle were not asked. In a prior analysis of mostly food secure African-American women of which the majority had personal vehicles for transportation but lived within a low food access area, food shopping behaviors were determined not by distance [43], but by food costs and quality [36]. Moreover, prior reports have shown the value of public transportation infrastructure and how access to transportation can impact nutritional food to insure adequate nutrition [17,44,45]. Within low-income-low- access food areas, public transportation routes are not planned or designed to facilitate access and availability to grocery food retailers [46], potentially causing hindrances time-wise or financially for those without personal transportation access. Agent-based modeling studies have shown a likely role for cities to invest in facilitating multiple routes of transportation (personal, shared ride, walking, cycling, public systems) to food retailer in low-income areas as a neighborhood-level intervention to optimize food shopping behaviors [46].

Further studies evaluating the role of transportation systems on food shopping behaviors are needed, as well as integration of transportation access and needs within diet behavior research, and specifically cooking intervention studies.

### Key results and directions of future work

Our finding that food shopping frequency served as a mediator between cooking frequency and diet quality, as measured by the Healthy Eating Index, among African-American adults at the population level has not been reported before to our knowledge. What could this mean for nutrition education and cooking interventions among African-Americans? Given the large mediation effect of more than 50%, it is likely that food shopping frequency is a key behavior of interest to amplify when seeking to improve diet quality among African-Americans. Future leverage of these results for community-engaged or nutrition education interventions that promote cooking frequency would be to include discussions on food shopping habits, measure major food shopping frequency, and to include in the curriculum education on meal habits that can increase food shopping frequency. Of note, based on our results measuring changes throughout an intervention may be important as major food shopping of less than one or more times a week was not associated with objective diet quality.

Compared to the use of geographic proximity measures, examination of food shopping behaviors provides a context to better understand how individuals interact with their available food environments. This context is especially pertinent for individuals experiencing food insecurity, as most residents within low food access areas travel outside of their neighborhoods to shop. Those who experience food insecurity may only have a selection of food retailers that have limited offerings and is therefore not consistent with conducting ‘major food shopping’. D’Angelo et al [28] reported that lower income adults use corner stores more frequently, retailers which are unlikely to be used for major food shopping; those shopping in corner stores are then more likely to procure unhealthy foods as compared to supermarket shoppers. To this point, in our analysis, individuals with food insecurity were more likely to have the lowest reported frequency of conducting ‘major food shopping’. In the absence of having data on the type of food retail stores available, we cannot state that this conclusively. But prior literature supports this inference in that those with food insecurity are more likely to purchase foods at smaller food retailers and not engage in ‘major food shopping’ [47]. This is important to consider as the question on food shopping frequency used in NHANES asked individuals to not include shopping trips when shopping for only a few items. Additionally, those who are food insecure may utilize food safety net services in their community (i.e. food banks) that were not measured in this study [48]. Consistent with the complexity of accurately capturing food shopping behaviors among those with food insecurity, food shopping frequency in our study was not a mediator between cooking and diet quality for those who experienced food insecurity or who lived in low income households. Future analyses and datasets may benefit from including factors that are reported as determinants of food shopping for those with food insecurity, such as food retailer type and utilization of community food services.

## Conclusions

Consistent with other studies, our analysis found that food shopping frequency among African-American adults is a behavior that is directly associated with a more optimal diet quality. We found this was not only true for diet quality over the course of an entire day, but also for dinner meals which often serve as the meal that is cooked at home. Relatedly, we found that food shopping frequency serves as a significant causal mediator between household cooking frequency and diet quality among African-Americans, but only for those who experience food security or have sufficient household income. For lower income and individuals experiencing food insecurity, food shopping frequency may not serve as a significant factor, and other factors to aid in diet quality optimization such as provision of transportation services to food retailers may be needed.

There are several limitations within our analysis that should be considered. One, cooking frequency was used as an indicator of behavior in this analysis, and it remains unknown if similar results occur when other indicators of cooking behavior are used. Two, our measurement of cooking is based on household frequency, and the interview respondents may not be the main person conducting the food shopping and cooking behaviors. This limitation also impacts our ability to determine sex-based differences in this analysis. Three, we were not able to determine geographic areas for the survey respondents, nor could we determine rural versus urban food environment settings in our sample. Four, although collected using a validated methodology dietary data within NHANES is based on self-report and subject to recall and reporting bias [49]. With regard to our use of mediation analysis, it is possible that there is a separate causal relationship between diet quality and food shopping (feedback model) that was not accounted for in our mediation model [50]. Lastly, the NHANES sample was surveyed during a time of economic crisis from the economic recession of 2008−09. Our results thus may indicate dietary choices and behaviors reflective of that economic context [51]. For example, employment shifts that occur during economic crises can led to a preference for ready-to-eat foods, especially if these foods are more convenient in terms of time costs [52]. Our study’s limitations may be mitigated in future NHANES analyses through inclusion of additional data elements in future studies. Future analyses to gain further insight into this finding could include asking about personal transportation and food retailer specific question items within NHANES. Further, the reintroduction of the cooking frequency question along with expansion to include meals beyond dinner could assist in providing further insight into how food shopping impacts diet quality and the diet behavior of cooking.

As public health efforts shift attention towards diet-related interventions to mitigate risk factors and disease severity of diet-related diseases, understanding associations between dietary behaviors and diet quality become increasingly important. Our findings suggest that among full or marginal food secure African-American adults in the U.S., there is a mediating effect of frequency of major food shopping on the relationship between household cooking frequency and dietary quality. Given the disproportionate number of African-Americans who may live in geographic regions where major food shopping may be a barrier, there is an important role for considering food shopping behaviors and how the food environment may impact major food shopping frequency when designing and cooking behavior interventions. Further, given the disproportionate number of African-American households that experience food insecurity, it is important for nutrition and diet behavior interventions within communities likely to experience food insecurity to include support for aiding food shopping or to measure barriers and facilitators that individuals utilize within adverse food environments. For interventions directed towards cooking and nutrition education through culinary skills, measuring food shopping frequency and building interventions that facilitate an increase in frequency (i.e. food retail store tours) may assist in optimizing diet quality. And within these interventions, assisting in providing instrumental support to food retail locations may become essential when recruiting individuals who are experiencing food insecurity.

## Supporting information

S1 Table Relationship between major food shopping frequency and food security status. (DOCX)

S2 Fig Stratified mediation between home cooking frequency and diet quality by poverty-income ratio. (DOCX)
