# Supplementary material for: Household cooking frequency and diet quality are mediated by food shopping behaviors among U.S. African-American adults: A NHANES analysis
Source: PLoS One. 2025 Jun 24;20(6):e0326481. doi: 10.1371/journal.pone.0326481 (PMC12186916; doi:10.1371/journal.pone.0326481)
Supplement: S1 Table — (DOCX) [file pone.0326481.s001.docx]

**Supplemental Table 1. Food shopping frequency categories by food security status**

| **Food Shopping Frequency Categories** | **Full Food Security** | **Marginal Food Security** | **Low/Very Low Food Security** |
| --- | --- | --- | --- |
|  |  |  |  |
| One or more times a week | 69.9 % (n=569) | 13.4% (n=97) | 16.7% (n=118) |
| Once every two weeks or a month | 60.0% (n=928) | 16.5% (n225) | 23.4% (n=324) |
| Rarely any | 65.4% (n=51) | 3.5% (n=2) | 31.0% (n=20) |

Chi-square analysis, Adjusted F 3.752, p = 0.018
