# Supplementary material for: Household cooking frequency and diet quality are mediated by food shopping behaviors among U.S. African-American adults: A NHANES analysis
Source: PLoS One. 2025 Jun 24;20(6):e0326481. doi: 10.1371/journal.pone.0326481 (PMC12186916; doi:10.1371/journal.pone.0326481)
Supplement: S2 Fig — (DOCX) [file pone.0326481.s002.docx]

**Supplemental Figure 1.**

**Highest Income to Poverty Ratio HEI-2010 Daily:**


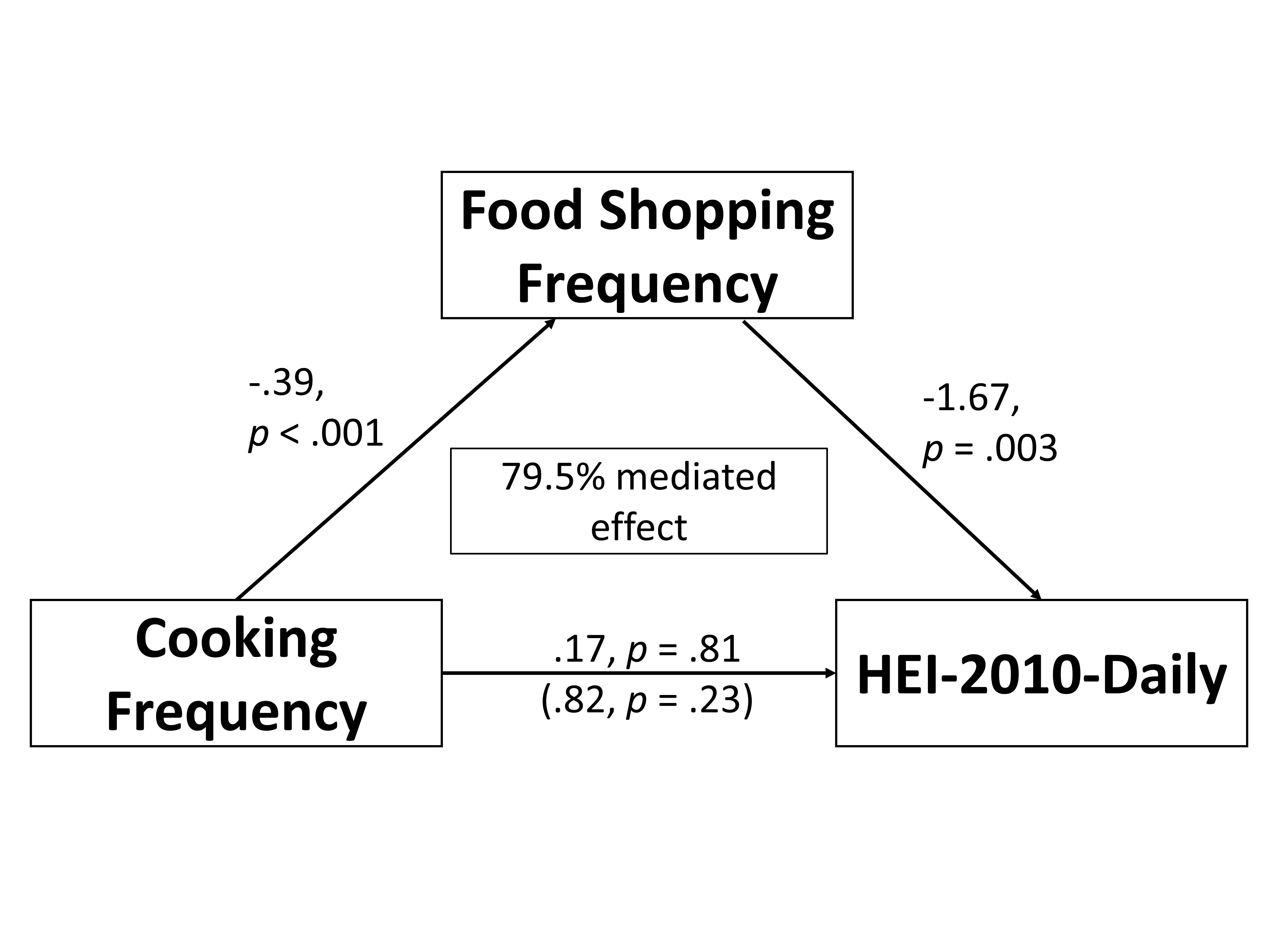


**Middle Range Income to Poverty Ratio HEI-2010 Daily:**


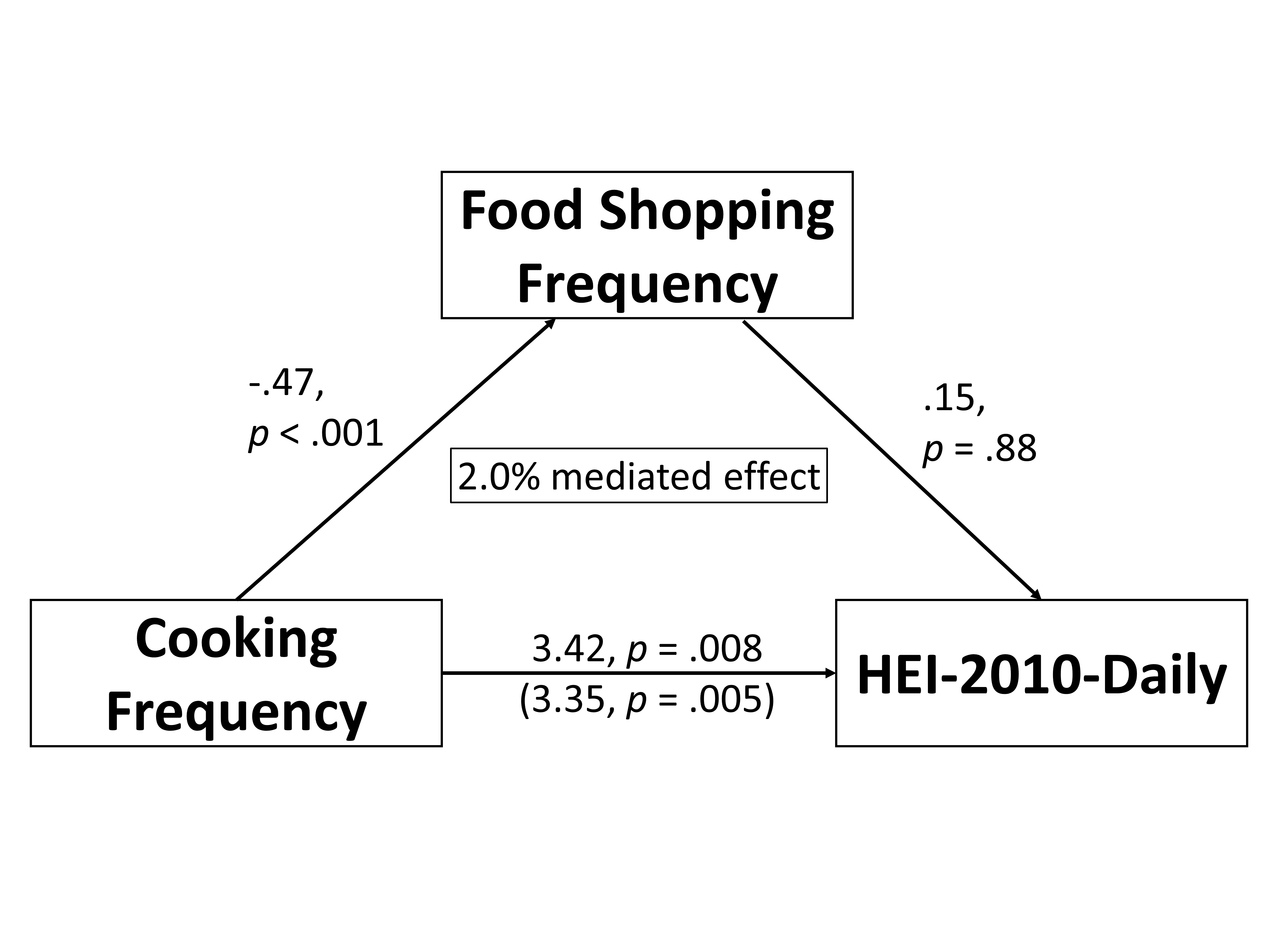


**Lowest Income to Poverty Ratio HEI-2010 Daily:**

**
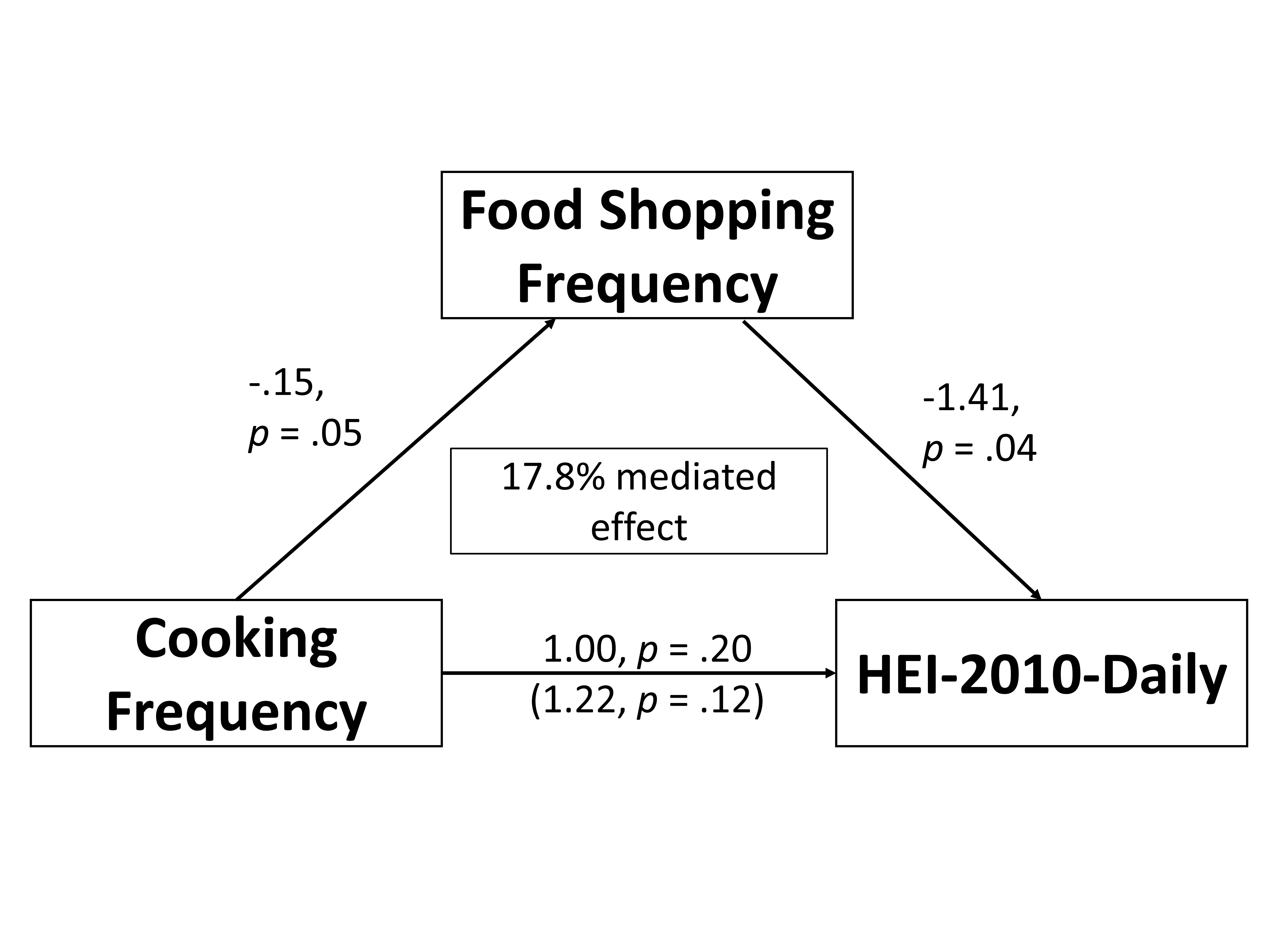
**
